# Impact of Polycyclic Aromatic Hydrocarbon Accumulation on Oyster Health

**DOI:** 10.3389/fphys.2021.734463

**Published:** 2021-09-10

**Authors:** Nin Gan, Leisha Martin, Wei Xu

**Affiliations:** Department of Life Sciences, College of Science and Engineering, Texas A&M University—Corpus Christi, Corpus Christi, TX, United States

**Keywords:** polycyclic aromatic hydrocarbons, oyster, bioaccumulation, environmental impact, host response

## Abstract

In the past decade, the Deepwater Horizon oil spill triggered a spike in investigatory effort on the effects of crude oil chemicals, most notably polycyclic aromatic hydrocarbons (PAHs), on marine organisms and ecosystems. Oysters, susceptible to both waterborne and sediment-bound contaminants due to their filter-feeding and sessile nature, have become of great interest among scientists as both a bioindicator and model organism for research on environmental stressors. It has been shown in many parts of the world that PAHs readily bioaccumulate in the soft tissues of oysters. Subsequent experiments have highlighted the negative effects associated with exposure to PAHs including the upregulation of antioxidant and detoxifying gene transcripts and enzyme activities such as Superoxide dismutase, Cytochrome P450 enzymes, and Glutathione S-transferase, reduction in DNA integrity, increased infection prevalence, and reduced and abnormal larval growth. Much of these effects could be attributed to either oxidative damage, or a reallocation of energy away from critical biological processes such as reproduction and calcification toward health maintenance. Additional abiotic stressors including increased temperature, reduced salinity, and reduced pH may change how the oyster responds to environmental contaminants and may compound the negative effects of PAH exposure. The negative effects of acidification and longer-term salinity changes appear to add onto that of PAH toxicity, while shorter-term salinity changes may induce mechanisms that reduce PAH exposure. Elevated temperatures, on the other hand, cause such large physiological effects on their own that additional PAH exposure either fails to cause any significant effects or that the effects have little discernable pattern. In this review, the oyster is recognized as a model organism for the study of negative anthropogenic impacts on the environment, and the effects of various environmental stressors on the oyster model are compared, while synergistic effects of these stressors to PAH exposure are considered. Lastly, the understudied effects of PAH photo-toxicity on oysters reveals drastic increases to the toxicity of PAHs *via* photooxidation and the formation of quinones. The consequences of the interaction between local and global environmental stressors thus provide a glimpse into the differential response to anthropogenic impacts across regions of the world.

## Introduction

As countries develop and human activities expand, so does the amount of waste produced and consequently, the amount of environmental pollution released. The rapid industrial growth of the 21st century has caused a demand for power satisfied primarily by petroleum that in 2020 has contributed to 34.7% of total energy consumption in the United States (United States Energy Information Administration, 2021), As a result, the United States has inadvertently become the largest source of polycyclic aromatic hydrocarbons (PAHs) in the environment either through incomplete combustion of coal, oil, petroleum, and wood (Abdel-Shafy and Mansour, 2016), or directly *via* oil spills such as the Deepwater Horizon spill (Reddy et al., 2012). The introduction of such chemicals to the environment does not come at no cost, and it is well-known that PAHs are toxic to humans (Abdel-Shafy and Mansour, 2016) and may constitute a possible health risk if bioaccumulated or otherwise present in food sources (Tongo et al., 2017; Rai et al., 2019; Abalaka et al., 2020).

A large body of research has taken interest in the environmental monitoring of the contaminants currently found in the environment by measuring PAH concentrations from the water column and in sediments (Yu et al., 2021). The inclusion of different media is very important to capture the distribution of contaminants because of the differential ability to accumulate contaminants. For example, the sediments at the bottom of the water column may act as a sink for contaminants since both metals and PAHs adsorb onto sediments (Jackim et al., 1977; Geffard et al., 2003), while organisms in the water column such as copepods ingest PAH-contaminated algae that later reaches the seafloor as fecal pellets (Arias et al., 2016). In fact, Guerra-García et al. (2021) considers sediment measurements to be preferable to seawater measurements in terms of characterizing the local pollutant levels due to the dynamic and variable nature of water in contrast to the more static ability of sediments to act as a storage for contaminants.

As for the organisms that live among the pollutants, chemicals enter the organisms’ bodies by diffusion, adsorption, or ingestion (Kelly et al., 2007; Arias et al., 2016; Metian et al., 2020; Shao and Wang, 2020), sometimes accumulating concentrations far exceeding that of either the water or sediments (Cortazar et al., 2008) although this is not always the case (Bustamante et al., 2012a; Hong et al., 2016; Vaezzadeh et al., 2019). Regardless, the use of bioindicators or organisms that accumulate contaminants to evaluate not only the levels but also the effects of environmental pollution is a useful method that likely provides a better representation of the anthropogenic impacts (Galloway et al., 2004). According to Zhou et al. (2008), biomonitoring reveals the integrated effects of the pollutants on the organisms by way of subtle biological changes that are not only sensitive due to the rapid onset of biological response but are also realized at contaminant levels that may be below detection limits due to chronic exposure to low doses. As a result, the bioaccumulation levels and responses in organisms such as algae, zooplankton, crustaceans, mollusks, fish, amphibians, and even marine mammals have been studied in aquatic and marine systems (Zhou et al., 2008; Cullen et al., 2019; Liu et al., 2019).

Gastropods and bivalves are two different classes of mollusks commonly used as bioindicators (O’Connor, 2002; Rittschof and McClellan-Green, 2005; Noh et al., 2018; Pham et al., 2020). Of these two, bivalves are potentially better suited for biomonitoring due to their sedentary lifestyle and filter-feeding. Oysters are especially of interest, due to their contaminant load often far exceeding their bivalve congeners (O’Connor, 2002; Leon et al., 2013; Wang and Lu, 2017; Liu et al., 2019) while displaying measurable signs of contaminant-induced stress. PAH exposure has been linked with increased antioxidant activity (Sarkar et al., 2017), altered immune system activity (Donaghy et al., 2010; Croxton et al., 2012), mutagenicity (Sarker et al., 2018), and larval abnormalities (Vignier et al., 2015) in oysters, which provide a long list of sublethal biomarkers with which to study the effects of such contaminants. Since oysters are of interest for human consumption, there are concerns over health risks due to their accumulation of toxic compounds (Hong et al., 2016; Liu et al., 2019; Wang et al., 2020), while the demand for the food source has been stimulating the growing oyster aquaculture industry (Botta et al., 2020). It is, therefore, no surprise that oysters have been extremely popular as bioindicators in recent decades. In this review, the contributions of various species of oysters (*Crassostrea brasiliana*, *C. rhizophorae*, *C. tulipa* (formerly *C. gasar*), *C. virginica*, *Magallana* (formerly *Crassostrea*) *angulata*, *M. belcheri*, *M. bilineata* (formerly *C. madrasensis*), *M. gigas*, *M. hongkongensis*, *Ostrea edulis*, *O. equestris, Saccostrea cucullata*, and *S. glomerata*) toward the bioaccumulation and toxicology of PAHs are presented.

## Oysters and PAHs

### Oysters as Biomonitor Organisms

Boening (1999) summarized that bioindicator organisms are suggested to: (1) be tolerant to the contaminant and accumulate contaminants without suffering mortality, (2) be either sedentary or have limited range of movement, (3) have a sufficient life span to allow for sampling over a broad range of time, (4) be abundant at the study site, (5) be of sufficient size for chemical analysis, (6) be hardy enough to maintain health through sampling and laboratory procedures, (7) be easy to sample and identify, (8) have high contaminant accumulation levels, and (9) be sensitive to changes in contaminant exposure. While not exhaustive, this list puts together the main concerns with the appropriateness of a bioindicator by highlighting ease of access, ability to reflect environmental change, and ease of robust scientific study. Further research efforts have added criteria for consideration as a bioindicator. (10) Bioindicators should have a low ability to metabolize contaminants (Hamzah-Chaffai, 2014) and (11) would be more useful if they had different life stages (Zhou et al., 2008). Organisms with a high ability to metabolize contaminants would in turn have a high efflux rate of any uptaken contaminants and may not accumulate detectable or appreciable levels of contaminant and lead to false-negative readings. Furthermore, Zhou et al. (2008) noted that having larva and adult stages provides the opportunity to study the effects of contaminants on multiple stages and the physiological processes of metamorphosis. As Boulais et al. (2018) demonstrated, the development and growth of the embryo stage of *C. virginica* were more sensitive to PAH-contaminated sediment than that of the veliger stage, and thus presents findings novel to studies on strictly adult oysters.

Oysters are palm-sized bivalve organisms, with an irregular shell, and a planktonic larval stage that metamorphoses into a sedentary adult life stage living attached to any hard substrate such as tree roots, pebbles and shells, or other oysters (National Research Council, 2004). As a result, some species such as *Magallana gigas* (formerly *Crassostrea gigas*) form vast oyster reefs that cover a wide area sufficient to transform entire benthic landscapes and habitats (Troost, 2010). The sessile nature of oysters results in the bioaccumulation of the contaminants in the immediate vicinity, while the wide-ranging reefs allow an equivalently wide sampling range that monitors the spatial distribution of point-source pollution. Due to the fact that oysters live amongst potentially contaminated sediments, and are filter-feeders that clear particles from large volumes of seawater each day (National Research Council, 2004), oysters are likely exposed to many times more contaminants than either non-filter-feeders or nekton that do not live near the sediments. Despite this, oysters continue to thrive due to their resilience to pollutants with several studies such as dos Reis et al. (2015) reporting no mortalities after exposing *C. brasiliana* to up to 1,000 μg L^–1^ phenanthrene (Phe) for up to 10 days, Perić et al. (2020) reporting no mortalities after exposing *O. edulis* and *M. gigas* to up to 100 μg L^–1^ copper and 50 μg L^–1^ cadmium for up to 4 days, and Aguirre-Rubí et al. (2018b) demonstrating that oysters stayed alive for as long as 10 days without water despite exhibiting possible contaminant-induced digestive gland atrophy and hemocytic infiltration of interstitial connective tissue. Thus their ability to survive despite the pollution while exhibiting measurable responses to it, combined with their distribution, living habits, and multiple life stages make oysters an exceptionally favorable bioindicator organism (Zhou et al., 2008).

### Characteristics of PAHs

PAHs are organic molecules made up of two or more aromatic hydrocarbon rings and have become a staple in the investigation of the effects of oil spills and coastal pollution near urban areas. Originating from sources such as petroleum (petrogenic) and the incomplete combustion of petroleum (pyrogenic), PAHs have become widespread across the globe, especially concentrated near urban centers (Laflamme and Hites, 1978; Wang et al., 1999). As such, they have become convenient proxies for all human activities that rely on fossil fuels. PAHs themselves are characterized by their low solubilities (Sangster, 1989) and are thus usually found adhering to small particles in either marine sediment (Vaezzadeh et al., 2019) or soil (Eker and Hatipoglu, 2019). Due to their semi-volatility, PAHs can be removed *via* evaporation or photooxidation (Karaca and Tasdemir, 2013; Eker and Hatipoglu, 2019), a process that could become problematic depending on the photoproducts. In general, photooxidation of PAHs yields reactive molecules, such as aldehydes and aromatic ketones known as quinones (Ehrenhauser, 2011), which are highly toxic (reviewed by Lee, 2003) and reminiscent of the compounds created during PAH metabolism (reviewed by Ertl et al., 2016). Therefore, PAHs are often regarded as carcinogenic and of great health risk due to their lipophilicity and ability to readily accumulate into body tissues (Abdel-Shafy and Mansour, 2016). Thus it is crucial to look into how PAHs have accumulated and their effects on the organism.

### Accumulation of PAHs in Oysters

Of the 16 PAHs recognized by the USEPA, all but the four smallest PAHs Naphthalene (Naph), Acenaphthylene (Acy), Acenaphthene (Ace), and Fluorene (Flu) have log *K*_ow_ > 4 (Sahu and Pandit, 2003) and are considered hydrophobic or lipophilic (La Guardia et al., 2012). Flu itself is on the borderline with a log *K*_ow_ = 3.96, whereas the widely accepted value of 4.18 (Sangster, 1989) makes Flu lipophilic. Therefore, among PAHs, solubility generally decreases with increasing molecular weight (Sahu and Pandit, 2003). Oysters tend to concentrate very high levels of PAHs, but the lipophilicity of the organic compounds affects the accumulation patterns. Since Geffard et al. (2003) found that the bioaccumulation factor (ratio of PAH concentration in the body to the PAH concentration in the sediments) of *M. gigas* larvae was negatively correlated with log *K*_ow_, a PAH is more likely to accumulate if it is more soluble.

#### Sizes of PAH Chemicals

In general, a 2–3-ring PAH is considered a low-molecular weight (LMW) PAH (Vaezzadeh et al., 2019; Pham et al., 2020; Wang et al., 2020) whereas a PAH having 4–6 rings is considered high-molecular weight (HMW), although Vignier et al. (2018) consider 3–6 rings as HMW and 2–4 rings as being soluble. Due to the difference in solubility, the HMW PAHs are more likely to be adsorbed to the sediment, especially if the sediment contains a large organic fraction (Baumard et al., 1999; La Guardia et al., 2012). In Dalian, China, HMW PAHs were found to accumulate throughout the year into sediments due to a calculated net flux from seawater to sediment (Hong et al., 2016). This could explain why in the water column near Hainan, China, 98% of the PAHs are LMW PAHs with 2–4 rings while 3-ringed (61%) and 4-ringed (28%) PAHs formed the majority of the sediment-bound PAHs (Wang et al., 2020). This highlights a shift toward HMW PAHs in the sediments. Vaezzadeh et al. (2019) found the mangrove sediments of Malaysia to be dominated by 5–6-ring PAHs, making up between 45–80% of the total PAHs found in the sediments. This in turn results in the increased bioavailability of the more soluble LMW PAHs (Baumard et al., 1999), and a greater concentration of LMW PAHs accumulated in the oyster. The PAHs accumulated in *M. belcheri* were reported to consist of at least 40% LMW PAHs in four out of five different mangrove sites around Malaysia (Vaezzadeh et al., 2019) despite the majority of HMW PAHs in the sediments. Resonating this pattern, the contaminants in both the *Magallana spp.* oysters and *Cymatium spp.* gastropods sampled from the Can Gio coastal wetland of Vietnam were enriched in 2–3-ring LMW PAHs, with the smallest PAH, Naph, dominating the PAH composition in oyster whole soft tissue (Pham et al., 2020). The marine oyster *O. equestris* predominantly accumulated the 3-ringed Phe in the Gulf of Mexico until major flooding of the Mississippi River occurred. However, molecular weight and bioavailability should not be taken as a law. Equal molecular weight pyrene (Pyr) and fluoranthene (Fla), exposed at roughly equal concentrations, still resulted in a soft tissue concentration of Fla exceeding 50% greater than that of pyrene in *S. glomerata* (Ertl et al., 2016). Increased uptake and/or retention of Fla or increased metabolism and elimination of pyrene potentially due to the observed upregulation of carbonyl reductase (CBR) were proposed to explain such discrepancies.

#### The Significance of Sediments

Despite increased bioavailability due to solubility, uptake of PAHs is not as simple as diffusion. Evidence suggests that the presence of sediments greatly increases PAH uptake. Exposure to PAH-contaminated sediments proved to be almost 100 times as toxic to *M. gigas* embryos than exposure only to the elutriate of those sediments (Geffard et al., 2001) suggesting that the PAHs in the sediments were more bioavailable than those in the water above in some way. Elutriates are essentially suspensions of “washed off” or decanted sediments and are synonymous with low or high-energy water attenuated fractions (HEWAF, LEWAF) that mix compounds of high log *K*_ow_ such as crude oil rather than sediments with water (Vignier et al., 2015). In the follow-up study by Geffard et al. (2002), unfiltered elutriate was tested against filtered elutriate. It was found that the unfiltered elutriates still contained over 10 times the total PAH concentration of the filtered elutriates, which was deduced to come from extremely fine suspended sediments > 0.7 μm minimum length. Larvae reared in the fine suspended sediments (unfiltered elutriate) bioaccumulated three times more PAHs in the 25% elutriate-dilution treatment (least dilution, most concentrated treatment) than did larvae reared in filtered seawater with no contaminants, but fed algae reared in the filtered elutriate. Since the filtered elutriate contained just one-tenth of the PAHs in the unfiltered elutriate, the fact that the larvae fed algae only contaminated with filtered elutriate was able to reach one third the PAH concentration of the larvae directly reared in the unfiltered elutriate provides additional support that ingestion is a highly significant mode of PAH accumulation. However, this only occurred at the highest concentration (25% elutriate). The other concentrations (1, 5, and 10% elutriate) still caused 50–100 ng/g dry weight of accumulation in the larvae reared in the unfiltered elutriate, while the larvae reared in clean seawater but fed contaminated algae accumulated close to nothing. Therefore, even though ingestion is an important mechanism for accumulation, it could be extremely limited when the contaminant levels in prey are very low, and the additional PAHs in the unfiltered elutriate may have been accumulated by the larvae through different means. Shao and Wang (2020) showed that even 60-nm nanoparticles would be ingested, while the suspended fine sediments in the unfiltered elutriate from Geffard et al.’s (2002) study were at least 700 nm (0.7 μm). Therefore, it could be the case that non-food particles are being consumed and contributing to the bioaccumulation of PAHs. A study has shown that a diet of particulate matter with less organic content is more likely to be rejected than particles more enriched in organic content by the cockle *Cerastoderma edule* (Iglesias et al., 1996), which could suggest that particles with enough organic matter may fail to be rejected and instead be consumed alongside food particles. Further research by Geffard et al. (2003, 2004) continues to reinforce the role sediments play in bioaccumulation, and suggests that resuspension of sediments, not unlike the action of elutriation, facilitate the uptake of PAHs and heavy metals.

#### Physiological PAH-Accumulation Affinity

The PAHs that are taken up, are then allocated to lipid-rich organs. Wang et al. (2020) break down the oyster anatomy into five portions: adductor, gill, gonad, hepatopancreas, and mantle. In other studies, the hepatopancreas is often referred to as the digestive gland (Bustamante et al., 2012b; Vaschenko et al., 2013; dos Reis et al., 2015; Shenai-Tirodkar et al., 2017; Aguirre-Rubí et al., 2018b; Perić et al., 2020; Shao and Wang, 2020) and the adductor is referred to as “muscle” (Bustamante et al., 2012b). The use of “soft tissues” is much more vague as it likely refers to gonads and mantle (Shao and Wang, 2020), non-gill tissue (Chan and Wang, 2019), or even all tissues combined and sometimes called either “total” or “whole” soft tissues (Aguirre-Rubí et al., 2018b; Sezer et al., 2018; Wang et al., 2020). In general, PAHs accumulated in the mantle or hepatopancreas, with gonad or gill next and adductor muscle last (Wang et al., 2020). Bustamante et al. (2012b) found that *M. gigas* exposed to the 4-ring PAH, pyrene (Pyr), for 24 h had the highest concentrations in the gills, followed by the hepatopancreas, then mantle, and then adductor muscle with twofold reductions at each step except for the last step in which the mantle contained 2.5 times more Pyr than the adductor muscle did directly after exposure. It was suggested that the mantle and especially gills may have high levels of PAHs simply due to their being the most exposed organs to the environment (Bustamante et al., 2012b) because, during a 15-day depuration period, some Pyr was retained in the mantle and hepatopancreas, but was removed from the gills rapidly. The high lipid content of the hepatopancreas and gonad may store lipophilic compounds such as PAHs (Wang et al., 2020), contributing to slower removal and increased bioaccumulation in those tissues. These findings led to the proposal that the mantle and hepatopancreas should be the target organs for biomonitoring data collection (Wang et al., 2020).

Differently sized organs of different species, therefore, result in differential accumulation within the same habitat conditions. Three different species of bivalves, the oyster *M. gigas*, mussel *Mytilus californianus*, and clam *Corbicula fluminea*, transplanted for 90 days within San Francisco Bay, United States, accumulated different levels of PAHs despite controlling for location and time (Oros and Ross, 2005). The oyster *M. gigas* accumulated the most out of the three species with total PAH ranging from 184–6,899 ng/g dry weight, while the mussel and clam accumulated 21–1,093 and 78–720 ng/g dry weight, respectively. Due to the high lipid content of oysters, this species is thought to be an excellent bioindicator for lipophilic contaminants such as PAHs, polychlorinated biphenyls (PCBs), and organochlorine pesticides (OCPs) (Fisher et al., 2000; Bustamante et al., 2012a; Leon et al., 2013; Trevisan et al., 2016; Aguirre-Rubí et al., 2018a) and even organic metals such as tributyltin (Alzieu et al., 1986; Higuera-Ruiz and Elorza, 2011) and methyl-Hg (Metian et al., 2020).

Once PAHs are taken up by the hepatopancreas, which is the detoxifying and xenobiotic-metabolizing center (Wang et al., 2020) akin to the human liver, many physiological mechanisms begin actively breaking down and detoxifying the potential carcinogen. In a process that will be discussed in the next section in greater detail, PAHs are reduced by stage I metabolic enzymes into water-soluble hydroquinones and later either excreted or conjugated by stage II metabolic enzymes into thio-conjugated o-quinones and subsequently excreted (Ertl et al., 2016). PAH-metabolism ultimately results in its depuration and effective detoxification. Some PAHs may be preferentially metabolized such as Pyr, Benzo[a]pyrene (BaP), and Benzo[a]anthracene (BaA) (Ertl et al., 2016), which may affect the ratios of PAH isomers such as BaP and BeP (Baumard et al., 2001). Since PAH ratios are often used as diagnostic ratios to track pyrogenic or petrogenic sources (Pie et al., 2015), preferential metabolism could lead to false readings and the calculation should be calibrated accordingly.

## The Effects of PAHs on Oysters

To assess the effects of a stressor, the researcher must identify biomarkers that would in theory react in some measurable way. In the case of chemical contaminants, most of the endpoints treat the compounds negatively as toxic and expect a weakening of physiology. The anatomy or histology could be directly observed to deviate from the norm, the physiological process could be expected to occur slowly or not at all, and mortality remains a viable effect. Other scientists take a more response-based approach and rely on the organism’s homeostatic mechanisms to alert any changes due to the contaminant. Such mechanisms could be detoxification processes that attack the contaminant or stress responses that either mitigate or repair damage caused by the contaminant. Still, other scientists may take a more abstract approach by measuring changes in gene expression that signal to the observer that the organism is in the process of either a detoxification process or stress response. In the following sections, a few basic biomarkers will be discussed and explained in the terms of an oyster, and a summary of the effects will be listed in Tables 1–3, according to whether the study was a laboratory-controlled test (Tables 1, 2) or an *in situ* observation of oysters collected from the field (Table 3).

**TABLE 1:** A summary of the results of the lab tests on the toxicities of PAHs is organized here, sorted by contaminant.

**Species**	**Contaminant (medium)**	**Biomarker**	**Effect**	**References**
*M. gigas*	Ant, Chr, IcdP, Phe (cyclohexane)	Immune response	Decreased hemocyte mortality	Bado-Nilles et al., 2008
*C. virginica*	BaP, Naph, Pyr (contaminated diatoms)	Immune response	Elevated agranular hemocyte numbers, Reduction in granular hemocytes, Decreased hemocyte mortality	Croxton et al., 2012
*M. gigas*	BbF (cyclohexane)	Immune response	Increased phenoloxidase activity	Bado-Nilles et al., 2008
*M. gigas*	DBahA (cyclohexane)	Immune response	Increased lysosomal presence	Bado-Nilles et al., 2008
*C. tulipa*	Flu (DMSO)	Detoxification response	Increase in CYP2AU1 transcripts only, no change in other CYP and GST transcripts in gills	Zacchi et al., 2019
*M. gigas*	Flu (DMSO)	Detoxification response, Histology	Increased CYP2AU2, GST, SULT transcripts in gills, No change in EROD, GST, MGST activity Elevated numbers of mucous cells in mantle	dos Reis et al., 2020
*M. gigas*	Naph (cyclohexane)	Immune response	Increased% non-specific esterase cells	Bado-Nilles et al., 2008
*C. brasiliana*	Phe (DMSO)	Detoxification response, Histology	CYP2AU1 levels increased in gills, mantle, digestive tract, Elevated numbers of mucous cells in mantle, Digestive tubular atrophy/thinning,	dos Reis et al., 2015
*C. brasiliana*	Phe (DMSO)	Detoxification response, Antioxidant response	Limited increase in CYP, SULT transcripts, no change in GST transcripts, No change in CAT, SOD transcripts	Zacchi et al., 2017
*C. brasiliana*	Phe (DMSO)	Stress response, Detoxification response, Antioxidant response, Oxidative damage	Limited inhibition of *hsp70* release, No difference in *hsp90* or *FABP* transcripts, Almost no difference in CYP, GST, SULT, SOD, CAT, GPx transcripts, No difference in CAT, GST, GPx, G6PDH, and MDA activities	Lima et al., 2018
*C. brasiliana*	Phe (DMSO)	Detoxification response, Antioxidant response	Reduced CYP and GSTΩ transcripts, Increased SOD, CAT, GST activity, Reduced GR activity	Lima et al., 2019
*C. tulipa*	Pyr (DMSO)	Detoxification response, Histology	Increased CYP, GST, SULT transcripts, EROD, MGST activity in gills Elevated numbers of mucous cells in mantle, Digestive tubular atrophy	Zacchi et al., 2019
*M. gigas*	Pyr (DMSO)	Detoxification response, Histology	Increased MGST activity in gills, Digestive tubular atrophy	dos Reis et al., 2020
*M. gigas*	Pyr, Flu (cyclohexane)	Immune response	Reduction in phagocytotic activity, No change non-specific esterase cell%, No change lysosome presence, Decreased hemocyte mortality	Bado-Nilles et al., 2008
*S. glomerata*	Pyr + Fla (contaminated rice flour)	Detoxification response, Immune response, Cell maintenance	Upregulation of laccase 1-like protein, carbonyl reductase, cytochrome p450 proteins Downregulation of aldo-keto reductase, B10-like, GST pi-class proteins	Ertl et al., 2016
*M. gigas*	PAH mixture—10 species (acetone)	Gamete health, Fertilization, Larval development	Fewer motile sperm, Reduced sperm swimming speed, Reduced linearity in swimming, Reduced fertilization success Delayed larval development	Jeong and Cho, 2005
*M. gigas*	PAH mixture—10 species (acetone)	Feeding, Metabolism	Reduced clearance rate, Reduced food absorption efficiency, Increased respiration rate, Increased ammonia excretion, No change in oxygen consumption vs ammonia excretion ratio, Reduced energy available for growth	Jeong and Cho, 2007
*C. virginica*	PAH mixture—24 species (DMSO)	Lysosomes	Lysosomal destabilization	Hwang et al., 2008
*M. gigas*	Diesel oil (WSF)	Immune response	No significant changes	Bado-Nilles et al., 2008
*M. gigas*	Heavy fuel oil (WSF)	Immune response	Reduction in phagocytotic activity, Decreased hemocyte mortality	Bado-Nilles et al., 2008
*C. virginica*	Crude oil from existing slick—Deepwater Horizon (DWH) (HEWAF), Dispersant corexit, Crude oil + dispersant (CEWAF)	Fertilization, Embryogenesis, Larval development	Gamete abnormality, Reduced fertilization success, Embryo abnormality, Reduced shell length, Extruded and granulated tissue, Mortality	Vignier et al., 2015
*C. virginica*	DWH oil slick (HEWAF), Dispersant Corexit, Crude oil + dispersant (CEWAF)	Larval development, Larval health and survival, Larval settlement	Reduced shell length, Starvation, sudden shell retraction, Narcosis, Mortality, Reduced settlement success	Vignier et al., 2016
*C. virginica*	DWH oil slick (HEWAF), Dispersant Corexit, Crude oil + dispersant (CEWAF)	Sperm viability, Fertilization, Sperm health and metabolism, Oxidative activity,	No change in survivorship or viability, Reduced fertilization success, Drop in mitochondrial membrane potential (increased metabolism), Increased acrosomal integrity, Reduced ROS activity	Volety et al., 2016
				
*C. virginica*	DWH oil slick (HEWAF)	Juvenile survival, Feeding, Histology, Tissue condition, Hemocyte health, Oxidative damage	No difference in mortality, Reduced clearance rate, starvation, Increased digestive tubule luminal surface area, digestive tubule atrophy and necrosis, prevalence of dilated lumens with reduced epithelial height, ulcers, hemocyte diapedesis in stomach and labial palps, Elevated numbers of hypertrophic mucous cells and mucus in alimentary tract, hyperplasia, Hemocyte apoptosis, Syncytia, Inflammation, Insignificant differences in lipid peroxidation	Vignier et al., 2018
*C. virginica*	DWH oil slick (HEWAF), Dispersant Corexit, Crude oil + dispersant (algae)	Larval development, Larval survival	Larval abnormality, Stunted growth, Mortality	Vignier et al., 2019
*C. virginica*	Crude oil—MC252 (mixed), Crude oil + SlickGone dispersant (CEWAF)	Juvenile survival Shell growth	Mortality, Reduced shell height	Schrandt et al., 2018
*M. gigas*	Crude oil—Brut Arabian Light (HEWAF), Dispersant FINASOL, Crude oil + dispersant (CEWAF)	Antioxidant response, Lysosomes, Histology	Increased laccase-type phenoloxidase, SOD activities, Lysosomal enlargement, Lipofuscin accumulation, Reduced neutral lipid content, Digestive tubule atrophy	Luna-Acosta et al., 2017

*This non-exhaustive list provides a collection of common end points used to study chemical toxicity and some of the effects of the contaminants that may manifest in a less controlled setting.*

**TABLE 2:** A summary of the results of the lab tests on the toxicities of contaminant mixtures with other pollutants in addition to PAHs is organized here.

**Species**	**Contaminant**	**Biomarker**	**Effect**	**References**
*C. virginica*	WAF of PAHs in sediment	Infection	Increased prevalence of *Perkinsus* infection	Chu and Hale. 1994
*C. virginica*	PAHs + heavy metals + pesticides in sediment suspension	Histology	Extensive neoplasia Renal cell tumors, Gill filament tumors, Gastrointestinal adenocarcinoma Neuroblastoma, Circulatory system tumors, Gonadal papillary lesions	Gardner et al., 1991
*M. gigas*	PAHs + heavy metals + miscellaneous contaminants in sediment or elutriate	Gamete health Fertilization Embryogenesis	No change in fertilization rate, Larval abnormalities	Geffard et al., 2001
*M. gigas*	PAH Heavy metals PAHs + heavy metals miscellaneous contaminants elutriate, contaminated algae	Embryogenesis Larval growth	Larval abnormalities, Mortality, Delayed larval growth	Geffard et al., 2002
*M. gigas*	PAHs + heavy metals (Cd, Cu, Zn) + miscellaneous contaminants in sediment or elutriate	Larval growth Metallothionein	Larval abnormalities, Metallothionein produced	Geffard et al., 2003
*M. gigas*	PAHs (various) + heavy metals (Cd, Cu, Zn) + miscellaneous contaminants in sediment or elutriate	Embryogenesis Larval growth	Delayed blastula formation, Larval abnormalities	Geffard et al., 2004
*C. virginica*	PAHs + heavy metals + miscellaneous contaminants in sediment suspension	Infection Immune function	No significant difference in neutral red uptake by hemocytes, Increased tetrazolium dye reduction, Increased ^3^H-leucine incorporation, Significantly increased rate of *Perkinsus* infection	Chu et al., 2002
*C. virginica*	PAHs + miscellaneous contaminants in sediment	Embryogenesis Larval development	Fertilization inhibition, Embryo abnormalities, Reduction in shell growth	Boulais et al., 2018
*C. brasiliana*	PAHs + heavy metals + miscellaneous chemicals in coal tar-based paint	Lysosomes	Lysosomal destabilization	Chiovatto et al., 2021

*The contaminant mixtures are either derived from sediments collected from contaminated sites or from anthropogenic chemical products. This non-exhaustive list provides a collection of common end points used to study chemical toxicity and some of the effects of the contaminants that may manifest in a less controlled setting.*

**TABLE 3:** The summary of the observations of the field collections reflecting the toxicities of heavy metals and PAHs is organized here.

**Species**	**Contaminant**	**Biomarker**	**Effect**	**References**
*S. cucullata*	Suspected PAH	Antioxidant activity, Detoxification response, DNA damage	Upregulation in CAT, SOD, Upregulation in GST, Greater number of DNA strand breaks	Sarkar et al., 2017
*S. cucullata*	Suspected PAH	DNA damage	Greater number of strand breaks	Sarker et al., 2018
*M. gigas*	Suspected PAH from oil spill	Immune function	Possibly reduced granulocyte count^*^ Possibly reduced phagocytic capacity^*^ Possibly reduced ROS production^*^	Donaghy et al., 2010
*M. gigas*	Suspected PAH from oil spill	Immune function	Impaired mitochondrial function in hemocytes Reduced number of competent phagocytes Reduced phagocytic capacity	Auffret et al., 2004
*C. virginica*	Suspected PAH and pesticide	Infection	Increased prevalence and intensity of *Persinsus* infection, but likely confounded	Wilson et al., 1990
*C. virginica*	Miscellaneous pollutants: heavy metals, PAH, PCB, pesticides (chlordane, DDT)	Immune function	Inconclusive changes in hemocyte activity, Elevated hemocyte density, Elevated mobile hemocyte numbers	Fisher et al., 2000
*C. virginica*	Miscellaneous pollutants: heavy metals, PAHs, PCBs, pesticides	Immune function	Elevated hemocyte numbers, Elevated mobile hemocyte number, Faster-moving hemocytes, Elevated lysozyme concentration in hemolymph, inconclusive increase in ROS by hemocytes,	Oliver et al., 2001
*C. brasiliana*	Suspected PAH, PCB, linear alkylbenzenes, fecal steroids, coliform bacteria, nitrogen, phosphorus	Oxidation damage	Elevated levels of reduced protein thiol and reversibly oxidezed protein thiol in the hemolymph	Trevisan et al., 2016
*C. rhizophorae*	PAHs, Ag, As, Pb, Cd	Flesh condition, Shell growth, Histology, Reproductive anomalies, Parasites, Inflammation, Stress on stress test	Increased soft tissue mass, Reduced shell weight, Digestive gland atrophy, Presence of edema, brown cell aggregates, Reduced gamete mass, Oocyte atresia, Increased presence of intracellular protists, Reduced ability to tolerate desiccation,	Aguirre-Rubí et al., 2018b

^*^The trends observed were not statistically significant. It is very difficult to attribute the effects to any particular cause, especially since none of the abiotic conditions are controlled in the field and that the oysters are under chronic exposure.

### Chemical Biomarkers: Antioxidant and Metabolic Enzymes

PAHs express their toxicity in the form of oxidative potential. Unlike heavy metals, the toxicity of PAHs comes from the intermediates formed during their breakdown process. As Ertl et al. (2016) summarized, hydrophobic PAHs are transformed during phase I metabolism of xenobiotics by cytochrome P450 (CYP) proteins and CBR into water-soluble hydroquinones that can be excreted. An increase in phase I metabolic protein transcripts and activity upon PAH-exposure thus suggest that a detoxification response has been initiated. Pyr has been found to cause significant initial spikes in the CYP proteins CYP3475C1, CYP2AU1, CYP2-like, and CYP356A transcripts in *C. tulipa* that dropped back to base levels by 96 h post treatment (Zacchi et al., 2019), while Flu exposure elicited a similar upregulation of CYP2AU2 in *M. gigas* (dos Reis et al., 2020). Potentially more lasting reactions were observed in *C. tulipa* individuals exposed to Flu, with significantly elevated levels of CYP2AU1 96 h post treatment, even though other CYP protein transcripts remained insignificantly changed (Zacchi et al., 2019). These results may be exhibiting a species-dependent response, in which “species” could ambiguously refer to either the organism or the PAH, or simple unreliability as biomarkers for PAH toxicity. Following phase I metabolism, the quinones (sometimes known as oxy-PAHs) produced are often even more toxic than their parent PAHs (Sarker et al., 2018; McCarrick et al., 2019) and present a serious electrophilic threat. During phase II metabolism, the conjugation of quinones with glutathione is catalyzed by glutathione S-transferase (GST) in order to detoxify and excrete contaminants *via* the mercapturate pathway (Cooper and Hanigan, 2018). Other pathways involve the conjugation of xenobiotics into sulfate esters by sulfotransferases (SULT) to facilitate excretion as well (Croom, 2012). Both enzymes experienced the similar, aforementioned short-term upregulation in *C. tulipa* and *M. gigas* exposed to Pyr and Flu, respectively (Zacchi et al., 2019; dos Reis et al., 2020). Overall, since the act of removal of PAHs causes its bioactivation as a toxicant, a constant supply of antioxidants is needed to prevent oxidative damage during PAH metabolism. Superoxide dismutase (SOD), catalase (CAT), and glutathione peroxidase (GPx), in addition to detoxification enzymes such as GSTs and SULT are thus potentially useful biomarkers to use when studying the effects of and tracking the accumulation of PAHs.

Indeed, levels of SOD, CAT, and GST in *S. cucullata* were found to be very closely correlated to total PAH concentrations from various sites in the Arabian Sea (Sarkar et al., 2017). In contrast, neither SOD, CAT, nor GST was found to be upregulated in *S. glomerata* exposed to dietary Pyr and Fla for 7 days, with GST ultimately being downregulated (Ertl et al., 2016). The phase I metabolic proteins, cytochrome P450 and CBR, were drastically upregulated, indicating active metabolism of the accumulated PAHs and reduction of the quinone intermediates, and it was proposed that preexisting SOD and CAT in the oysters may have been sufficient in clearing the reactive oxygen species (ROS) formed during this process. In addition to cytochrome P450 and CBR, the third group of phase I metabolic enzymes, aldo-keto reductases (AKR), specializing in metabolizing aldehydes and ketones was downregulated instead. This possibly suggests a preferential pathway of CBR reduction (Ertl et al., 2016) or a lack of need for AKRs in PAH breakdown. As for the downregulation of GST, it should be noted that such ambiguous phenomenon was observed in *O. edulis* and *M. gigas* by Perić et al. (2020) following the exposure of heavy metals Cu and Cd, and that antioxidant or metabolic enzymes should not be exclusively relied upon as biomarkers of PAH exposure. The lack of any change in CYP, GST, SULT, SOD, CAT, and GPx transcripts and/or activity in the gills of *C. brasiliana* after Phe exposure (Lima et al., 2018) followed by the downregulation of CYP, GSTΩ and increase in SOD, CAT, and GST activity in the same experiment with *C. tulipa* (Lima et al., 2019) further promotes caution regarding the reliability of these biomarkers. It is interesting to note that the oysters observed in the field under chronic exposure (Sarkar et al., 2017) returned the more expected results in expression of antioxidant and metabolic proteins, while the oysters under the acute, controlled laboratory exposures exhibited much less expected and more complicated results (Ertl et al., 2016) and may be related to the intertwining of PAH metabolism and toxicity. For example, acutely-exposed oysters may rely on pre-existing SOD, CAT, and GST as needed, since PAHs only exhibit toxicity when transformed, while those chronically-exposed could never maintain a reservoir of SOD, CAT, and GST and must constantly produce them.

### Mutagenicity and Carcinogenicity

In addition to oxidative damage on tissues such as lipid peroxidation due to PAHs, DNA damage was observed in a few studies. PAHs have long been recognized in many studies as a cancer-causing agent in marine systems (Le Goff, 2017). Besides lipids, nucleic acids are a susceptible target of oxidative attack. DNA damage is often characterized by DNA strand breaks or unwinding (Sarkar et al., 2017; Sarker et al., 2018), and by problems during DNA replication involving micronuclei formation (McCarrick et al., 2019). *S. cucullata* collected off the west coast of India were found to contain DNA strand breaks seemingly proportional to the amount of PAH pollution at each site (Sarkar et al., 2017). The follow-up study by Sarker et al. (2018) expanded the previous study by including multiple abiotic parameters and heavy metal concentrations to rule out confounding variables and found that PAHs contributed to at least 87% of the variation in DNA integrity. Lab results confirm that the effects of BaP on the loss of DNA integrity is dose-dependent (Sarker et al., 2018) and support the hypothesis that PAHs as mutagens in oysters.

Left unchecked, mutagens may cause cancerous tumors and lesions in several different tissues in oysters over chronic exposure. 13.6% of *C. virginica* exposed to contaminated sediment off the coast of New York at a suspended concentration of 20 mg/L for 30–60 days developed tissue neoplasms including renal cell tumors, water tube and gill filament tumors, gastrointestinal adenocarcinoma, neuroblastoma, circulatory system tumors, and gonadal papillary lesions (Gardner et al., 1991). Although, the sediments contained heavy metals, pesticides, and PCBs in addition to PAHs, the concentration of accumulated non-PAHs in oysters never exceeded 8× the pre-treatment control levels, whereas PAHs accumulated between 6 and 60× the pre-treatment control levels with most being over 20× pre-treatment levels. Due to a lack of available literature on PAH-induced neoplasia in mollusks and the difficulty of attributing etiology in field studies by the aforementioned confounding variables, the reliability of cancerous lesions as a biomarker for PAHs is uncertain thus far. Although neoplasia has been observed in fish from contaminated habitats in many studies (reviewed by Le Goff, 2017), many of the confounding variables remain in the pathogenesis of the neoplasia, such as viruses which are known to cause a wide variety of neoplasia including hemic neoplasia. Longer-term laboratory-controlled experiments in zebrafish (*Danio rerio*) have revealed a link between petroleum compounds and carcinogenic effects especially on the liver (Larcher et al., 2014), which could equate to similar results if tested on mollusks.

### Diseases

Should the prevalence of neoplasia be due to increased viral infection, alteration of immune function by PAH exposure may be a possible cause. There is evidence to suggest that PAHs are toxic to hemocytes. For example, *M. gigas* exposed to dietary PAHs on spiked diatoms displayed increases and decreases in different types of hemocytes (Croxton et al., 2012). More specifically, Pyr and BaP exposure increased the proportion of agranular hemocytes while reducing the granular hemocyte proportion in part due to mortality. Hemocyte apoptosis was also observed in *C. virginica* juveniles that were exposed to a HEWAF of crude oil (Vignier et al., 2018). This could be the cause of reduced phagocytic capacity, which was reported to occur in *M. gigas* collected in areas affected by oil spill (Auffret et al., 2004). Without distinguishing between the types of hemocytes, Xie et al. (2017) recorded a net decrease in hemocyte count, and the proportion of mobile hemocytes, those involved in phagocytosis, in the pearl oyster *Pinctada imbricata* (formerly *Pinctada martensii*) from Pyr exposure. On the other hand, apparent increase in immune system activity was found in *M. gigas*, that exhibited decreased hemocyte mortality, increased phenoloxidase activity, lysosome count, and the% of non-specific esterase cells upon exposure to a wide variety of PAHs (Bado-Nilles et al., 2008). *S. glomerata*, having been exposed to Pyr and Flu for 7 days, differentially expressed many unique pattern recognition receptor proteins (Ertl et al., 2016) involved in detecting and neutralizing pathogens, including peptidoglycan recognition proteins, C1q domain-containing proteins, toll-like receptors, c-type lectins, c-type mannose receptors/macrophase mannose receptors, galectin, gram-negative bacteria binding protein, scavenger receptor, 2′–5′-oligoadenylate synthase 1, and fibrinogen-related proteins. In the field, exposure to an oil spill off the coast of Korea was associated with a possible reduction (not statistically significant) in granulocyte population and phagocytic capacity in *M. gigas* (Donaghy et al., 2010), while chronically exposed *M. gigas* measured to have elevated PAH tissue concentrations expressed immunotoxicity in the form of hemocytes with impaired mitochondrial function and a 50% reduction in phagocytic function (Auffret et al., 2004).

It follows then, that lab experiments show an increase in both disease transmission and susceptibility with increasing concentration of a combination of PAHs and heterocyclic compounds (Chu and Hale, 1994; Chu et al., 2002). The prevalence of *Perkinsus marinus* increased both in oysters inoculated with the parasite and in oysters placed nearby without direct inoculation when treated with a less-diluted water-soluble fraction of contaminated sediments from the Elizabeth River near the Chesapeake Bay in the United States (Chu and Hale, 1994). Due to the use of only the water-soluble fraction, this result was achieved using the more soluble low molecular weight PAHs and heterocyclic compounds. In practice, however, this result is much more ambiguous. The prevalence and intensity of *P. marinus* were not found to significantly correlate with total PAH body-burden but associated with urban land use instead (Wilson et al., 1990). Many other factors were suggested to confound this relationship. For example, high latitude lower temperatures, and low salinities were all associated with reduced infection load. It was proposed that colder conditions reduce reproductive seasons, which in turn reduce efflux of PAHs resulting in greater body burden while being less susceptible to *P. marinus* (Wilson et al., 1990). Little could be said about the connection between PAHs and infection with *P. marinus*, and its correlation thus far seems to be coincidental.

### Histology and Physiology

Besides changes in immune function, additional physiological consequences arise in oysters exposed to PAHs. The algae clearance rates of both *M. gigas* adults and *C. virginica* spat algae clearance rates were found to be reduced in individuals exposed to a PAH mixture and HEWAF of Deepwater Horizon slick oil, respectively (Jeong and Cho, 2007; Vignier et al., 2018). Shell closure, a physiological defense against unfavorable water conditions, was blamed for the reduced feeding among the contaminated oysters (Vignier et al., 2018). Once the valves shut, feeding ceases and the oyster begins to decline in health and experience symptoms reminiscent to that of starvation, especially due to the lack of partially-digested food particles in the alimentary tract (Vignier et al., 2018). Degradation of the alimentary canal was observed, with HEWAF-treated juvenile *C. virginica* exhibiting digestive tubule atrophy and necrosis, luminal dilation, ulcers, inflammation of the stomach and labial palps, hypertrophic and overrepresented mucous cells in the lumen along with a truncation in the height of luminal epithelial cells (Vignier et al., 2018). Similar digestive tubule atrophy was also observed in *C. tulipa* and *M. gigas* exposed to Pyr or crude oil HEWAF, while an increase in mantle mucosal cells was seen in *C. tulipa* exposed to Pyr and *M. gigas* exposed to Flu (Luna-Acosta et al., 2017; Zacchi et al., 2019; dos Reis et al., 2020). The increase in mucous cells in the lumen was accompanied by excess mucus as well as hemocyte diapedesis in the alimentary tract as a possible strategy to expel xenobiotics (Luna-Acosta et al., 2017; Vignier et al., 2018). Digestive gland edema was also found in wild *C. rhizophorae* from a contaminated site in Colombia (Aguirre-Rubí et al., 2018b). Yet, despite the inflammation and erosion of the digestive tract, mortality rates among spat remained low and insignificantly changed due to exposure to the HEWAF of crude oil.

Lysosomal health has been traced in response to PAH-related stress as well. Lysosomes are organelles filled with hydrolytic enzymes and helpful to the detention of xenobiotics (reviewed by Hwang et al., 2008; Wang et al., 2018). Lysosomal enlargement in digestive cells occurred in crude oil HEWAF-exposed *M. gigas* (Luna-Acosta et al., 2017), potentially indicating storage of accumulated oil or PAHs. The lysosomes in hemocytes, however, were not only observed to increase in number in *M. gigas* exposed to DiBenzo[a,h]Anthracene (DBahA) (Bado-Nilles et al., 2008), but also experience membrane permeability in a process known as lysosomal destabilization in *C. virginica* exposed to a mixture of 24 PAHs and *C. brasiliana* exposed to a variety of chemicals including PAHs in coal tar-based paint (Hwang et al., 2008; Chiovatto et al., 2021). This membrane breach may result in the release of cathepsins into the cytoplasm (reviewed by Wang et al., 2018) that ultimately could be responsible for the hemocyte apoptosis recorded by Vignier et al. (2018) in juvenile *C. virginica* exposed to crude oil HEWAF. Lastly, since the exposure of *M. gigas* to the chemical dispersant FINASOL^®^ caused only lysosomal biomarkers to be affected, and did not cause the additional symptoms such as antioxidant activity and digestive tubule atrophy associated with Brut Arabian Light oil HEWAF and CEWAF exposure (Luna-Acosta et al., 2017), changes to lysosomal activity may be very sensitive endpoints to consider for PAH-exposure.

### Gametic and Larval Health

Due to rapid turnover time, high quantities, and high sensitivity to contaminants, the early life stage developmental milestones (embryo, larvae) are useful biomarkers for PAH toxicity assessment. Millions of larvae could be produced rapidly, only taking roughly 24–48 h from zygote to D-shaped larva. Abnormal larval shape is the most common endpoint, described by His et al. (1997) for *M. gigas* larvae as possessing morphological aberrations in the form of a convex hinge, indented shell margin, incomplete shell, and/or a protruding mantle. Such abnormalities are potentially the result of rapid shell closure that occurs so quickly that, like juvenile oysters, the larvae are left with a protruding mantle or velum (Vignier et al., 2016). The subsequent starvation of the larval oyster could then explain the reduced shell length, larval abnormality, stunted growth, and mortality observed in larval *C. virginica* exposed to the HEWAF of oil from the Deepwater Horizon spill (Vignier et al., 2015, 2016, 2019). Later-term pediveliger larvae of settlement age exposed to the same HEWAF also experienced a drastic reduction in spatfall or settlement success across all concentrations with no dose-dependency (Vignier et al., 2016). The chemically-enhanced water accommodated fraction (CEWAF) of the oil using the dispersant Corexit exhibited a clear, almost linear, dose-dependency of settlement success, despite potentially greater access to the PAHs (Vignier et al., 2016). Therefore, the dramatic drop in settlement success in the HEWAF assays were either due to a PAH-exclusive effect on physiology or the coating of settlement surfaces with PAHs. The former seems not to be the case, since the presence of the dispersant alone usually resulted in extreme toxicity, often far greater than the HEWAF alone (Vignier et al., 2016, 2019; Volety et al., 2016).

When exposed to PAH-contaminated sediment elutriate during pre-fertilization, *M. gigas* sperm showed no significant changes in health and fertilization success (Geffard et al., 2001). These findings were corroborated by Volety et al. (2016), who found that neither the survivorship, viability, nor acrosomes of the *C.* virginica sperm cells were negatively affected by exposure to the HEWAF of crude oil. In fact, it was found that ROS activities had unexpectedly dropped, which was explained by greater metabolic activity occurring and the active detoxification of the ROS. However, exposure of both sperm and oocyte of *C. virginica* to a HEWAF of crude oil or sediment elutriate resulted in a significant reduction in fertilization success (Vignier et al., 2015; Volety et al., 2016; Boulais et al., 2018). This may suggest that the eggs are susceptible to the toxicity of PAHs. Post-fertilization exposure to unfiltered elutriates produced from contaminated sediment for 24 h resulted in a totality of abnormal larvae (Geffard et al., 2001). The larvae accumulate PAHs so quickly that elongated exposure time did not significantly change the number of abnormal larvae (Geffard et al., 2004). Having contact with the sediment was many times more potent than being in contact with only the unfiltered elutriate (Geffard et al., 2001, 2003, 2004), corroborating the notion that sediments provide a powerful vehicle for the accumulation of contaminants. Other modes of entry, such as ingestion of algae reared in contaminated elutriate, exhibited similar results to non-filtered elutriate (Geffard et al., 2002). Based on the Effective Concentration for 50% abnormality (EC_50_) values for gametes (267 μg/L, Vignier et al., 2015) and embryos (342 μg/L reported by Vignier et al., 2015; 22.4 g/L reported by Boulais et al., 2018), the gamete is more sensitive than the embryo, and the embryo is more sensitive than the larval stage is to environmental contaminants (Boulais et al., 2018).

## Abiotic Conditions

Oysters of the order *Ostreida* are a group of hardy bivalves globally distributed in intertidal and shallow subtidal regions within sheltered bays, mangroves, or estuaries with freshwater input (Gosling, 2003). This subjects them routinely to wide temperature and salinity fluctuations through which the oysters survive. Temperature and salinity ranges may be at least as wide as 3–35°C and 5–40 ppt for *M. gigas* (Bayne, 2017), and 5–25°C and 16–34 ppt for *O. edulis* (Hutchinson and Hawkins, 1992; Bayne, 2017). However, optimal ranges for growth and reproduction are much narrower, with ranges constricted to approximately 11–34°C and 22–35 ppt for *M. gigas*, and 15–20°C and 30–32 ppt for *O. edulis* (Shatkin et al., 1997; Wiltshire, 2007; Bayne, 2017), beyond which stress response systems activate to maintain homeostasis. Such responses are critical for the survival of oysters and other bivalves under extreme and/or prolonged environmental change.

The environment is also not a static system, especially under the human influence by which rising *p*CO_2_ levels are expected to reach 538 ppm by 2,100 under the Relative Concentration Pathway of 4.5 W/m^2^ of radiative forcing (RCP4.5), the current most conservative climate change scenario predicted by IPCC (2013). At this rate, the global mean surface temperature is expected to increase by at least 1°C with global ocean surface pH dropping below 8 (IPCC, 2013). Under the worst-case scenario RCP8.5, the global mean surface temperature may increase by up to 3°C or more with global ocean surface pH falling below 7.8. These projections imply that organisms will potentially be subject to chronically elevated temperatures in addition to ocean acidification. Increasing frequency of heavy precipitation events and tropical cyclones, deemed to occur more likely than not in the late 21st century (IPCC, 2013), will cause drastic short-term changes in salinity in estuarine and bay systems whether it be massive dilutions due to extreme precipitation and flooding (Edmiston et al., 2008; Munroe et al., 2013) or salinity rises due to storm surge (Huang et al., 2014). Mass die-offs of 55–90% of oysters have already been recorded within the Gulf of Mexico due to prolonged periods of reduced salinity as low as 0 for several weeks (Edmiston et al., 2008; Munroe et al., 2013), so it is clear that additional abiotic factors affect oyster health, and may compound onto concurrent PAH-burden.

### Salinity and PAHs

The solubility and bioavailability of PAHs, especially that of LMW PAHs, increases with decreasing salinity (Shukla et al., 2007), which may result in the increased uptake of PAHs as demonstrated in fish including tilapia (*Oreochromis mossambica*, formerly *Tilapia mossambica*), rainbow trout (*Oncorhynchus mykiss*), and mummichog or Gulf killifish (*Fundulus heteroclitus*) at lower salinities (Ramachandran et al., 2006; Shukla et al., 2007). However, such straightforward effects are not always observed, especially if other abiotic factors are involved, as evidenced by the complete lack of correlation between salinity and tissue-concentration of PAHs in oysters collected from different sites (Bebianno et al., 2007). When bivalve mollusks experience osmotic shock, they often close their valves (Kurihara, 2016) to limit water movement, and then attempt to osmoregulate by deamination of free amino acids (reduce internal concentration) or decomposition of proteins (increase internal concentration) (reviewed in Pourmozaffar et al., 2020). Withdrawal and shell closure has also been associated with starvation since the oyster is no longer feeding (Vignier et al., 2018), which may further complicate additional burdens by placing tighter limits on energy reserves for more prolonged exposure times. Furthermore, lower salinities have been associated with a host of negative effects on bivalves such as lipid peroxidation in the Mediterranean mussel (*Mytilus galloprovincialis*) and littleneck clam (*Ruditapes philippinarum*) (Velez et al., 2016; Freitas et al., 2017), reduced shell length and thickness of the blue mussel (*Mytilus edulis*) (Maar et al., 2015), reduction in the body cavity index (ratio of the flesh mass to the internal cavity volume) of *C. virginica* (Heilmayer et al., 2008), reduced growth rate and size of both *O. edulis* and *O. lurida* larvae (Robert et al., 1988; Lawlor and Arellano, 2020), and decreased phagocytosis activity coupled with increased hemocyte mortality in several bivalves (reviewed by Pourmozaffar et al., 2020). This could mean that hypoosmotically-weakened oysters are highly susceptible to PAH-compounded health consequences resulting from the increased accumulation of more-soluble PAHs.

In a thus far rare experiment testing the effects of salinity on Phe exposure on *C. brasiliana*, strictly increasing bioaccumulation was not observed with decreasing salinity (Zacchi et al., 2017). Instead, the intermediate salinity (25) accumulated the highest amount of Phe (88.4 μg/g dry weight) followed by low salinity (10, 81.5 μg/g dry weight) and then high salinity (35, 72.0 μg/g dry weight) (Zacchi et al., 2017). It may be possible that non-optimal salinities (low and high) have inhibitory effects on the bioaccumulation of PAHs, which would almost serve as a protective measure. However, low salinities still carry the burden of hypoosmotic stress or other mechanisms that negatively affect the health of oysters. The intermediate amount of Phe accumulated at the lowest salinity was associated with elevated CYP isoforms (CYP2AU1 and CYP2-like1) after 24 h of exposure with high levels of CYP2-like1 remaining after 96 h when compared to the control (Zacchi et al., 2017). As CYP proteins are involved in stage I metabolism of PAHs, this shows an increased effort to detoxify Phe at low salinities, possibly due to increased toxicity. As a result, the antioxidant enzymes CAT-like, SOD-like, and two GST proteins GSTm-like and GSTΩ-like were upregulated in the low salinity treatment compared to the two higher salinity treatments as expected. Other than oxidative stress; however, Phe did not seem to inhibit osmoregulation activities and gene expression since amino acid-metabolizing proteins were upregulated with no impact from Phe (Zacchi et al., 2017).

Animals other than oysters have showed increased sensitivity to PAHs at lower salinities as well. The LC_50_ of the HEWAF of crude oil was found to be significantly reduced, reflecting increased mortalities, at the lower salinities in all three animals tested, grass shrimp (*Palaemon pugio*, formerly *Palaemonetes pugio*), sheepshead (*Cyprinodon variegatus*), and mud snail (*Ilyanassa obsoleta*, formerly *Tritia obsoleta*) (DeLorenzo et al., 2021). In fact, there was a clear positive relationship between salinity and LC_50_. Mussels (*Mytilus galloprovincialis*) collected at different sites along the south coast of Portugal where salinity varied from 4–34.9 psu exhibited cytochrome P450 levels consistent with what levels would be expected based on the tissue-concentration of PAHs (Bebianno et al., 2007). GST levels from PAH metabolism were found to vary inversely with salinity, which reflects the increased amount of stress at lower salinities. The pattern of GST levels occurred regardless of the tissue-concentration of PAHs, which possibly indicated that the detoxification activity responded to the compounding of low salinity on PAH burden rather than the increasing PAH accumulation at lower salinities. Such apparent changes in toxicity due to reduced salinity were not observed in a number of studies that varied abiotic factors during the exposure of fish to a water attenuated fraction of weathered oil (Serafin et al., 2019; Simning et al., 2019; Allmon et al., 2021). Additionally, Schrandt et al. (2018) found that oil (MC252)-exposed juvenile *C. virginica* showed a clear interaction between oil exposure and salinity on the survivorship and shell growth. Both survivorship and shell growth diminished with oil exposure. Surprisingly, however, the effects of oil appeared to be exacerbated by the moderate salinity level (18) vs. the low salinity level (8) (Schrandt et al., 2018), despite the moderate salinity level (15–18) being recommended as the optimal salinity for *C. virginica* (Wallace, 2001). It was suggested that short-term exposure to oil is mitigated by salinity changes due to the shell closure reflex, thus preventing further exposure to oil as well. A discrepancy between short-term and long-term exposures alongside abiotic conditions must then be accounted for in the results from lab experiments, especially in this case in which salinity changes confound rather than compound the effects of contaminant exposure.

### Temperature and PAHs

Temperature plays a huge role in controlling biological events in oysters such as reproduction and spawning (Bayne, 2017) and subsequent larval swarming (Maathuis et al., 2020). Over small ranges, the temperature has little effect (Matoo et al., 2013), in part due to the wide thermal tolerances of most oysters. But where temperature fluctuations are greater, physiological effects often scale monotonically with temperature, and to a greater degree in contrast to the relationship with salinity. Increasing temperature is not only significantly correlated with growth rate and calcification (Robert et al., 1988; Harney et al., 2016), but also heat duress, during which heat shock proteins (hsp) are upregulated (Yang et al., 2016; Nash and Rahman, 2019; Nash et al., 2019), oxidative stress in both juvenile and adult oysters (Moreira et al., 2017), and reproductive problems marked by cell apoptosis in the gonads (Nash and Rahman, 2019; Nash et al., 2019). Considering the negative effects associated with exposure to high temperatures, the effects could compound with additional PAH exposure. Sampling oysters during the wet and dry season in areas contaminated with PAHs offers a glimpse into how temperature may alter the effects of PAHs. The prevalence of digestive gland oedemas in *C. rhizophorae* from Colombia was only observed in significantly high numbers in oysters taken from one contaminated site during the wet season, but became ubiquitous across all sites during the dry season (Aguirre-Rubí et al., 2018b). In addition, the number of reproductive anomalies and presence of *Nematopsis spp.* parasites was elevated in oysters sampled during the wet season from what was considered to be one of the more contaminated sites in Colombia, and even more exaggerated during the dry season (Aguirre-Rubí et al., 2018b). These examples of the effects of pollution becoming more visible or only visible during the dry season provide evidence toward compounding effects of temperature and PAH toxicity. Since it has been found that the pearl oyster *Pinctada imbricata* (also formerly *Pinctada radiata*) accumulated a significantly greater amount of Phe at 28 vs 24°C (Jafari et al., 2021—in review), it is very possible that the greater PAH-burden would also cause more deleterious effects.

Dolphin fish (*Coryphaena hippurus*) larvae exposed to weathered oil HEWAF exhibited very clear dose-dependent effects on the abnormal fluid accumulation in the heart and cardiac output (Perrichon et al., 2018). Edema size, sinus venosus-yolk mass gap, and incidence of intrapericardial hematomas increased with increasing total PAH concentration, whereas heart rate and stroke volume both decreased. Increasing temperature caused a greater sensitivity in stroke volume to total PAH concentration, or in other words, an accelerated effect of the total PAH concentration on the reduction of blood flow. However, the effect of total PAH concentration on the sinus venosus-yolk mass gap was reduced by the increase in temperature (Perrichon et al., 2018), meaning that greater concentrations of PAHs lost some of their potency in ability to cause abnormal swelling. This could be due to a compounded effect of heat stress and oxidative stress from PAH-body burden that caused toxicity to approach its peak lethality, but at the same time, elevated temperature failed to cause more damage. In the aforementioned trio of grass shrimp (*Palaemon pugio*), sheepshead (*Cyprinodon variegatus*), and mud snail (*Ilyanassa obsoleta*), exposure to the HEWAF of Louisiana Sweet crude oil significantly reduced the LC_50_ of each species when treated at 32°C rather than 25°C (DeLorenzo et al., 2021), apparently lending support to increased toxicity of PAHs at higher temperatures. Unfortunately, available literature on this topic is sparse at the time so there is little else that can be said about the synergistic effects of PAHs and temperature change on a marine organism’s health.

However, heavy metal toxicity is very well-studied and often causes similar effects such as oxidative stress and oxidative damage on oysters (Jo et al., 2008; Shenai-Tirodkar et al., 2017; Alexander et al., 2019). Arsenic (As) exposure in *M. gigas* larvae contracted the optimal ranges of both temperature and salinity (Moreira et al., 2018). This could be interpreted as either the heavy metals causing non-optimal abiotic conditions such as increased temperature to be even more damaging than before or that those oyster larvae outside their optimal temperature and salinity ranges are more susceptible to As poisoning. The former case seems to be more supported, as it was also shown that cadmium (Cd) exposure in *C. virginica* caused a sharp increase in mortality at the high temperature of 28°C when individuals not subject to Cd exposure seemed to tolerate the high temperature quite well showing no difference in mortality at any of the different temperature treatments (20, 24, and 28°C) (Lannig et al., 2006). Complementarily, lead (Pb) exposure in mussels (*Mytilus galloprovincialis*) at higher temperatures appeared to cause a significant inhibition of the release of the antioxidant enzymes SOD, CAT, and GPx (Freitas et al., 2019). Thus, it may be possible to infer that exposure to certain PAHs may also reduce an oyster’s ability to tolerate a wide temperature range by causing more oxidative damage than expected outside the optimal range.

Indeed, such reductions in tolerance could be the case, as the release of *hsp70* and fatty acid-binding proteins (*FABP*) seemed to be somewhat inhibited (although not statistically significant) in the oyster *C. brasiliana* with Phe exposure at all temperatures tested (18, 24, and 32°C) (Lima et al., 2018). However, even though the accumulation of Phe increased at 32°C compared to at 24 and 18°C, the effect of temperature itself was so great that any consequence of Phe exposure was largely overshadowed. Virtually no differences in both detoxification enzyme and antioxidant enzyme transcript levels and activities were observed between Phe-exposed oysters and uncontaminated oysters at each temperature (Lima et al., 2018). In addition, greater oxidative damage was not observed by Freitas et al. (2019) in the aforementioned Pb-exposed mussels despite the apparent inhibition of release of antioxidant enzymes. It seems that the effects of temperature on PAH toxicity have no obvious pattern. It also may be the case that when interactive effects are observed, it is more likely that the PAH exposure adds to the negative effects of temperature, rather than the temperature changing the toxicity of PAHs. It is clear that although some evidence of the potential for compounding effects between temperature and contaminant exist, the system is dynamic, the synergistic effects are difficult to capture, and that further study is extremely necessary.

### PAHs During Ocean Acidification

There is little doubt that ocean acidification due to rising atmospheric *p*CO_2_ levels will have a negative effect on bivalves such as oysters. Mussels and likely other bivalves calcify their shells by controlling the pH directly underneath the shell, creating a region of elevated pH and CO_3_^2–^ concentration that promotes CaCO_3_ precipitation (Ramesh et al., 2017). This vital activity causes bivalves to be sensitive and often negatively affected by changes in external pH. A CO_2_-mediated drop in seawater pH has been shown to reduce larval shell growth, cause abnormalities in the larval shell, and ultimately result in a significantly reduced survivorship in many different bivalves including scallops (*Pecten maximus*) and oysters (*M. gigas*) (Andersen et al., 2013; Barros et al., 2013; Timmins-Schiffman et al., 2013; Frieder et al., 2017). Due the potentially increased energetic demands of maintaining homeostasis under acidification, the Dynamic Energy Budget model predicts reductions in shell length, flesh weight, and reproductive capacity in adult mussels (Ren et al., 2020). Consistent with this model, stunted growth rate was observed in both juvenile clams (*Mya arenaria*) and oysters (*M. gigas*) (Beniash et al., 2010; Zhao et al., 2018), featuring reduced shell mass, soft-tissue mass, and shell strength. Under such an energy-draining abiotic factor, the ability to detoxify contaminants and prevent or repair any oxidative damage is likely to be greatly challenged.

In one of the only currently available pieces of literature on the effects of PAHs on oysters under acidification conditions, Lima et al. (2019) demonstrates that *C. tulipa* upregulates SOD, CAT, and GST activities despite a reduction in glutathione reductase (GR) activity due to Phe exposure. While reducing the pH to 7.0 and 6.5 and did not cause any change in antioxidant activity besides a slight drop in *GR* activity in the oysters not subject to Phe contamination, the same drastic pH change resulted in a downregulation of all three enzyme activities down to levels no longer significantly different from that of the uncontaminated oysters. Therefore, low pH may have inhibitory effects on the ability of oysters to detoxify xenobiotics and break down ROS.

There is some evidence that lower pH levels may also have inhibitory effects on the immune response of mollusks. While BaP exposure appears to have reduced granulocyte count and phagocytosis rates in the blood clam *Tegillarca granosa*, the low pH treatments (7.8 and 7.4) appear to compound the effects of BaP and further reduce granulocyte activity (Su et al., 2017). Furthermore, the immune system signaling pathways were negatively affected by the downregulation of toll-like receptors (TLR1, TLR4, TLR6), myeloid differentiation primary response protein (MyD88), TNF receptor-associated factor 6 (TRAF6), transforming growth factor beta-activated kinase 1 (TAK1), inhibitory κB kinase α (IKKα), and nuclear factor kappa B (NF-κB). Together, these immunomodulating proteins recognize pathogens and signal various immune system pathways that initiate an inflammatory response (Kawasaki and Kawai, 2014). Although the toll-like receptor protein 2 (TLR2) and TAK1-binding protein 2 (TAB2) were upregulated, all other immunomodulatory proteins were downregulated significantly not only by BaP exposure, but also by reduced pH. In fact, reduced pH compounded the effects of BaP and caused a significant and even further downregulation in the oysters exposed to BaP at low pH (7.4) compared to either expression level due to BaP exposure or low pH alone.

Again, the well-studied, toxic effects of heavy metals may be considered to make predictions about the effects of PAHs under acidified conditions. *M. gigas* oysters exposed to Cd at a the low pH of 7.6 suffered the most oxidative stress in the digestive gland, with elevated SOD, GST, GPx, and lipid peroxidation even though the slightly more modest acidified condition (pH 7.8) saw slightly greater accumulation of Cd (Cao et al., 2018). In a follow-up study, Cao et al. (2019) found that *M. gigas* individuals exposed to both Cu and low pH (7.6) exhibited several toxic effects in addition to greater oxidative stress and lipid peroxidation than in individuals subject only to Cu contamination. Those oysters suffered gill damage in the form of increased prevalence of vacuolization, cilia erosion, and gill lamina hypertrophy, which could partially be attributed to the increase in accumulation of Cu at the lowest pH tested (7.6). At the same time, the oysters within the low pH, Cu-exposure treatment group had reduced algae clearance rates, respiration rates, and flesh condition beyond that of the oysters receiving only Cu-exposure. This inevitably would result in reduced energy reserves with consequences related to the dynamic energy budget. Evidence for that scenario was found in *S. glomerata* exposed to Cu at elevated levels of *p*CO_2_ (1,000 μatm). This group of oyster individuals maintained physiological activities at levels not significantly different from unexposed individuals; however, the eggs produced by the females were smaller with less lipid content (Scanes et al., 2018). This was hypothesized to be due to a shift in energy allocation from maternal investment to homeostatic maintenance. Surprisingly, the embryos fertilized from the more poorly-developed eggs developed normally; however, such larvae may be at a much greater disadvantage if they were to grow in nature away from the protective laboratory conditions. In summary, increased acidity of the water generally compounds the negative effects of the existing contaminants. With new findings that Phe accumulates in greater quantities in *Pinctada imbricata* at reduced pH levels (Jafari et al., 2021—in review), it is highly likely that acidification would cause even greater stress in oysters burdened by chronic exposure to PAHs.

### PAH Photo-Toxicity

One interesting property of PAHs is their potential to be photo-toxic. Not unlike the bioactivation of PAH toxicity during its metabolism, photo-oxidation of PAHs in natural sunlight not only creates ROS immediately, but also breaks them down into toxic quinones (Lee, 2003), which are not only capable of producing ROS but are also more soluble (Ertl et al., 2016) and likely more bioavailable. Lee (2003) reviews what is considered the primary mechanism underlying PAH photo-toxicity: PAHs release a free radical upon sunlight exposure that reacts with oxygen thereby creating ROS. The photo-products such as anthraquinone, benzo[a]anthraquinone, phenanthrenequinone are often significantly more toxic than their parent PAHs were (Lee, 2003). The water-attenuated fractions of weathered oil treated with UV light were shown to be significantly more toxic than untreated oil WAFs (Finch et al., 2016; DeLorenzo et al., 2021). In particular, Finch et al. (2016) demonstrated and isolated UV-light as the effective component of sunlight that induces phototoxicity. The effective concentration at which 10% of the *C. virginica* larvae or sperm were found to be abnormal (EC_10_) dropped drastically with increasing intensity of UV light exposure on the water-attenuated fraction. Different oils gave different values of EC_10_ (Finch et al., 2016), which suggests that oil composition and the presence of compounds that are more likely to be photo-toxic play an important role in the potential photo-toxicity of a given oil film. This discrepancy between oils was observed by Pelletier et al. (1997), in which one of the fuel oils with lower PAH composition, did not display photo-toxicity in contrast to the other three oils tested. The lethal concentrations (LC_50_) of the oils to brine shrimp (*Americamysis bahia*) translated to the UV-treated oil as being drastically more toxic than the fluorescent light-treated oil (control), ranging from 4× to upward of 80× more toxic, with similar jumps in toxicity being recorded for the survival and development of the dwarf surf clam (*Mulinia lateralis*) (Pelletier et al., 1997).

**FIGURE 1:**
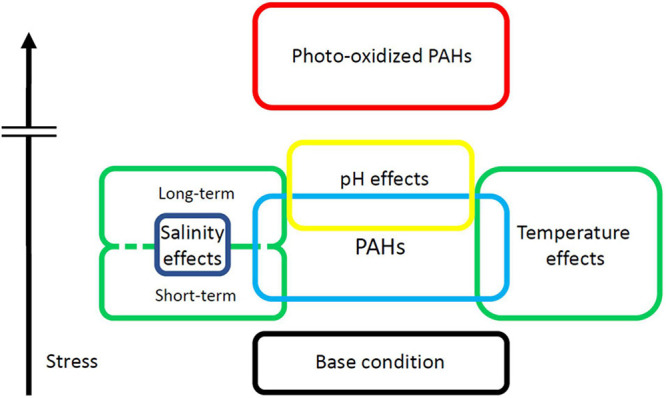
The PAH-induced stress hierarchy. PAHs induce bodily stress above that of normal uncontaminated conditions. Salinity and Temperature may add to or mitigate the stress of PAH-burden. Photo-oxidized PAHs are far more toxic than their parent PAHs, and their increased potential to cause harm is represented by the break in the vertical bar.

UV-treatment of individual PAHs boasted an exaggerated 10 s of 1,000 s of times more toxic than fluorescently-treated PAHs (control) were tested to be (Pelletier et al., 1997) and is graphically represented in Figure 1. The toxicity of Pyr and BaP reached similar levels after UV-exposure, needing only 5 μg/L of either PAH to cause complete failure in the larval cohort, whereas doses of 1 μg/L of either PAH saw UV-exposure as the difference between having approximately 80% abnormal (UV-exposure) or 80% (no UV-exposure) normal D-shaped larvae (Lyons et al., 2002). In the mussel (*Mytilus galloprovincialis*), four PAHs: Pyr, Phe, Fla, and chrysene (Chr) were tested (Okay and Karacik, 2008). Phe and Chr were found to be especially photo-toxic as UV exposure yielded an approximately 60–80% drop in filtration rate (Phe only) and neutral red retention (measure of lipid membrane integrity) in the hemocytes. In contrast, neither the feeding rate nor the neutral red retention dropped significantly after UV exposure in both Pyr and Flu (Okay and Karacik, 2008), but it is clear that PAH-accumulation under UV exposure has massive potential for harm compared to simply accumulating PAHs by themselves.

## Conclusion

The oysters of the genera *Crassostrea*, *Magallana*, *Ostrea*, and *Saccostrea* play a vital role in our understanding of the distribution and potency of pollutants that result from anthropogenic activities. Oysters are a model bioindicator organism capable of accumulating large concentrations of contaminants while being easy to collect data from, and providing data that is easy to conceptualize. They provide valuable insight into how PAHs affect marine ecosystems. The negative consequences of the bioaccumulation of PAHs are likely to be felt by all species, but it is our choice of bioindicator that reveals this information. Oysters have revealed that PAHs cause oxidative damage, DNA strand breaks, dampened immune systems, reduced fertilization rates, larval abnormalities, changes in body mass, and fierce metabolic and antioxidant responses, while abiotic conditions such as temperature, salinity, pH, and especially UV-light could increase these effects. Across the many studies, some biomarkers such as disease prevalence or antioxidant and detoxification response have some ambiguity in the results, and caution should be applied during their interpretation. All possibilities of exposure duration, stage of response, and confounding factors should be considered carefully. Due to this caveat, it is important to realize that the health problems observed in wild oysters may not necessarily reflect the stressor of interest due to the existence of a myriad of other contaminants and abiotic conditions and their synergistic effects, and the chronic nature of the exposure. The environment is extremely complex and wrought with confounding factors. This makes it nearly impossible to pinpoint the exact cause without a controlled lab test. However, wild oysters will never be exposed to controlled conditions in the wild, and thus, more synergistic effects must be studied. At this time, most synergistic effects of coexposure to changes in abiotic conditions and PAHs are thus far poorly explored, especially with more ambiguous interactions such as between temperature changes and PAH exposure. Further study should take more synergistic effects involving abiotic conditions into consideration, and may consider using less common biomarkers involving histology to observe tissue damage and neoplasia. Alternatively, novel biomarkers could be explored as well, such as the heart rate of oyster cardiac muscle cells and the gene expression of stress-related proteins pioneered by Xu et al. (2018).

## Author Contributions

NG did the literature review and drafted the manuscript. WX edited and finalized the manuscript. All authors contributed to the article and approved the submitted version.

## Conflict of Interest

The authors declare that the research was conducted in the absence of any commercial or financial relationships that could be construed as a potential conflict of interest.

## Publisher’s Note

All claims expressed in this article are solely those of the authors and do not necessarily represent those of their affiliated organizations, or those of the publisher, the editors and the reviewers. Any product that may be evaluated in this article, or claim that may be made by its manufacturer, is not guaranteed or endorsed by the publisher.
